# Perceived Effectiveness of Differing Health Warning Label Messaging Strategies among Adults in the Republic of Georgia: One Size Does Not Fit All

**DOI:** 10.3390/ijerph15102221

**Published:** 2018-10-11

**Authors:** Cailyn Lingwall, Eric Nehl, Marina Topuridze, Lela Sturua, Nuka Maglakelidze, Carla J. Berg

**Affiliations:** 1 Department of Behavioral Sciences and Health Education, Rollins School of Public Health, Emory University, Atlanta, GA 30322, USA; cailynlingwall@gmail.com (C.L.); enehl@emory.edu (E.N.); 2 National Center for Disease Control, Tbilisi, Georgia; topuridzemarina@gmail.com (M.T.); lela.sturua@ncdc.ge (L.S.); N.maglakelidze@ncdc.ge (N.M.); 3 Winship Cancer Institute, Emory University, Atlanta, GA 30322, USA

**Keywords:** tobacco control, tobacco control policy, tobacco use, public health policy, low- and middle-income countries

## Abstract

*Background*: While pictorial health warning labels (HWLs) are evidence-based, the different messaging strategies are understudied. *Methods*: We analyzed 2014 national survey data from 1163 Georgian adults to examine: (1) perceived effectiveness of pictorial vs. text-only HWLs; (2) pictorial HWL themes; and (3) correlates of perceived effectiveness of different pictorial themes. Participants were randomized to evaluate the effectiveness of either Set A or Set B of HWLs (each contained half pictorial, half text-only). *Results*: All but 2 pictorial HWLs were perceived as more effective than text-only. Factor analyses identified one factor among Set A (“benign”) and two in Set B pictorial HWLs (“benign”, “gruesome”). Among Set A pictorial HWLs, correlates of greater perceived effectiveness included being female, rural residence, not having children, and nonsmoker status. Among smokers, correlates included being female and unmarried, fewer smoking friends, and higher quitting importance. Among Set B, 43.8% rated gruesome pictorial HWLs more effective, 12.9% benign more effective, and 43.4% equally effective. Correlates of perceiving benign more effective included fewer smoking friends and higher income. Among smokers, lower income predicted gruesome being perceived as more effective; fewer smoking friends and higher quitting importance predicted perceiving benign as more effective. *Conclusion*: A variety of pictorial HWL strategies should be used.

## 1. Introduction

The tobacco epidemic is one of the biggest public health threats globally, killing more than 7 million people a year [1]. Low- and middle-income countries (LMICs) are disproportionately impacted by tobacco-related deaths [1], with approximately 80% of the world’s 1.1 billion smokers living in LMICs [1]. Smoking rates in former Soviet Union countries are among the highest in the world [2]. The Republic of Georgia is one LMIC in this region [3] with high smoking rates; as of 2016, 57% of adult males and 7% of adult females were current smokers [4].

For more than a decade, the World Health Organization’s Framework Convention on Tobacco Control (WHO FCTC) has provided a major impetus for all countries to adopt comprehensive policies to counter the global tobacco epidemic [5,6]. The core demand reduction provisions in the FCTC are contained in Articles 6–14 and address pricing and taxation, secondhand smoke exposure, product regulation, and particularly relevant to the current study, packaging and labeling [5,6]. Nearly 90% of the world’s population lives in the 181 countries that have ratified the FCTC, underscoring the importance and potential impact of implementing these evidence-based strategies [7]. Unfortunately, and perhaps related to high smoking prevalence in many LMICs and particularly former Soviet Union countries, adoption and enforcement of FCTC provisions has lagged in many of these countries [2].

The health warning label (HWL) policy included in the FCTC is one promising area for tobacco control. According to the FCTC Article 11 guidelines, HWLs should cover at least 50% of the package but not less than 30% [6]. These recommendations are based on robust evidence that text-only HWLs are largely ineffective [8] and that pictorial HWLs are easily understood, have broad reach, demonstrate population impact on smoking uptake and cessation, and are cost-effective [6].

While Georgia has lagged in implementing FCTC articles, legislation effective May 2018 will progressively advance tobacco control. One component of the legislation is to implement pictorial HWLs, beginning in January 2019. Thus, evidence is needed regarding the impact of differing pictorial HWLs in Georgia in order to inform these images and messaging strategies. Initial research to inform pictorial HWL messaging strategies before implementation in many contexts began with studies of the perceptions of the population to which they would be applied [9,10,11,12]. Subsequent research then evaluated the actual consequences of pictorial health warning labels once implemented in certain countries/contexts [11,12,13,14,15,16,17,18,19,20]. One limitation of the latter two approaches is that the vast majority (if not all) of these studies were unable to (or did not aim to) specify the impact of the differing HWL messaging strategies, as most countries/contexts used a variety of HWL messaging strategies. In the context of Georgia’s current status, research suited to the phase of informing their pictorial HWL messaging strategies should thus examine individual perceptions of various strategies. Such research will likely be relevant to other countries in this region and more broadly.

The Elaboration Likelihood Model (ELM) is a relevant model for understanding the impact of HWLs, as it provides a lens for developing persuasive risk communication to change attitudes and, ultimately, behavior [21]. ELM posits that there are two routes to attitude change, the central and the peripheral routes [22]. ELM suggests that, when individuals with prior motivation to process a particular message encounter another message containing similar arguments or in cases where the message is particularly salient, ‘central processing’ is more likely to occur [23]. Attitudes formed through the central route are more likely to be resistant to change and are a better predictor of future behavior [22]. In contrast, if someone with less motivation to process a particular message encounters a message with similar arguments, they are more likely to rely on ‘peripheral cues’ to assess that message [23]. Peripheral cues may include social cues, or the perceived expertise and credibility of the source [22]. Attitudes formed through central processing following exposure to a message are more likely to be stable compared to those from peripheral processing [23]. In short, this model suggests that personal relevance and involvement with the message are the most influential factors in how one processes a message [22].

The ELM is a highly relevant model for examining pictorial HWL effectiveness. While pictorial HWLs have been shown to be effective in general, the impact of images and messaging differs based on the content. For example, graphic images displaying pathology of illness have been shown to be more effective than abstract images [24]. In fact, benign or abstract pictorial warnings that do not provoke emotional reaction (e.g., images depicting a skull or burned fingertips) may be less effective than text-only labels [24]. However, when faced with pictorial HWLs vividly depicting the threat of tobacco-related illness, smokers may have an “optimistic bias,” unrealistically believing they are not susceptible to illness [25].

The ELM also suggests that individual characteristics may impact one’s perceptions of different messaging strategies. Indeed, individual smoking-related characteristics are associated with impact of pictorial HWLs. For example, pictorial HWLs may increase non-smokers’ intentions to avoid initiation, particularly among those who already have high self-efficacy in refusing tobacco [26]. Pictorial HWLs alone, however, may not be effective among smokers who lack confidence to quit if the HWL does not address smokers’ self-efficacy relative to quitting [26]. Smoking-related social factors may also influence reactions to pictorial HWLs; for example, pictorial HWLs may lead non-smokers to help friends and family quit smoking [27].

Given that smoking prevalence often differs by sociodemographics in various countries [28,29], many of the correlates of smoking are also associated with reactions to pictorial HWLs. For example, while HWL content does not have to target youth or adults to have an emotional impact [12], younger populations may believe that they can quit smoking before facing illness and perceive that HWLs showing long-term health issues are not relevant [30]. Other evidence suggests that younger people may perceive HWLs effective that depict the cosmetic consequences of smoking including rotten teeth, premature skin aging, or wrinkled skin [12]. In addition, female smokers are more likely than male smokers to consider quitting after looking at a pictorial HWL [31], women of reproductive age are more responsive to pictorial HWLs about pregnancy [32], and women are more likely than men to perceive pictorial HWLs with non-gruesome content, such as those depicting a human experience related to smoking, as more effective [33]. Moreover, people in rural areas have a lower general awareness of all forms of tobacco information, including HWLs [34]. In terms of educational status and literacy, pictorial HWLs may be an educational tool in communicating risks to low-literacy populations who may not understand text-based HWLs [13,35].

Given the aforementioned literature and gaps in current knowledge, this study was guided by the ELM and examines HWL messaging strategies and their impact among Georgian adults. Specifically, this study examined: (1) perceived effectiveness of pictorial vs. text-only HWLs; (2) themes emerging from various pictorial HWLs recommended by the European Union; and (3) correlates of perceived effectiveness of different themes of pictorial HWLs among adults in the Republic of Georgia. In relation to the latter, potential individual correlates of perceived effectiveness of HWL messaging strategies included sociodemographic variables and tobacco use and related factors (e.g., importance of quitting, confidence in quitting, social influences).

## 2. Materials and Methods

### 2.1. Study Protocol and Participants

The current study is an analysis of a cross-sectional national household survey of Georgian adults conducted from February to May 2014. The study was approved by Emory University IRB (IRB00070852) and National Centers for Disease Control and Public Health IRB in the Republic of Georgia (IRB#2013-027a). This population-based survey included adults aged 18–64 who lived in the Republic of Georgia and were able to read Georgian or English. The survey was administered in ten regions as well as the capital. The sampling frame was selected using the 2002 census data, the most recent data available. A multi-stage cluster sampling design was used to identity participants. Stratification was performed by region, with each region divided into urban and rural strata and yielding 22 total strata. Among the 22 strata, 122 clusters were formed to provide at least nine interviews per cluster. Sample size was calculated proportionately by the number of households in the regions [36].

The “random walk” method was used to select households in the cluster. The “KISH method” used in the WHO’s STEPS surveys was then used to select eligible participant within the households [37]. According to the KISH method, all eligible participants from the household were first ranked by age in decreasing orders, with males followed by females. Next, participants were selected using the KISH table, which identified the last digit of the household as well as number of eligible participants [37]. Out of the 1539 visits performed, 1295 total households contained eligible adults for the survey. A total of 1163 adults participated in the survey, yielding an 89.8% response rate [36].

### 2.2. Measures

The survey assessed information on sociodemographic factors, tobacco use history and influences, and perceptions of multiple tobacco policies. The current analyses focused on perceptions of HWLs. Below, we highlight each of the measures included in the analyses.

#### 2.2.1. Predictors

Sociodemographic variables. Participants were asked to report sociodemographic factors which included their age, sex, setting of residence (urban/rural), years of education, total monthly household income in GeL (Georgian Lari), employment status, relationship status, and number of children in the home.

Tobacco use and related factors. All participants were asked if they currently smoke tobacco on a daily basis, less than daily, or not at all. For analysis, those who reported daily smoking and less than daily were classified as “current smokers” [38]. Current smokers were asked to report the number of days smoked in the past 30 days, number of cigarettes smoked per day [38], how important quitting smoking was to them, and how confident they were that they could quit smoking, respectively, on a ten-point scale (0 = not at all to 10 = extremely) [39]. To assess social influences regarding tobacco, all participants were asked to report the number of friends out of their five closest who smoked.

#### 2.2.2. Outcome

Perceived effectiveness of HWLs. The HWLs included in the survey were drawn from those recommended by the European Union [40]. In order to examine how persuasive the pictorial versus text-only versions of the messages were, participants needed to examine one or the other (pictorial or text-only) of each message. Additionally, having one subset of individuals evaluate all pictorial or all text-based messages could have biased the results. As such, the pictorial HWLs were first randomized to be included in Set A or Set B (and then the text-only version of the HWL were presented in the opposite set). Participants were then randomized to evaluate Set A or Set B in order to compare responses to pictorial versus text-only HWLs.

Perceived effectiveness was assessed by asking: “On a scale of 1–9, where 1 is ‘not at all’ and 9 is ‘extremely,’ please indicate how effective this message might be in motivating smokers to quit smoking or preventing people from starting smoking?” Participants were randomized to receive either Set A HWLs or Set B HWLs (see Table 1), which included half pictorial images and half text-only messages.

### 2.3. Data Analyses

All statistical modeling was conducted using SPSS 23.0 (IBM, Armonk, NY, USA), with significance set at *p* < 0.05. Descriptive analyses were conducted to characterize the study sample (Table 2). Preliminary analyses were then conducted to examine differences in sociodemographic characteristics as well as tobacco use in participants who completed Set A versus Set B (which indicated that only employment status was statistically different, such that Sample A had a larger proportion who were unemployed, *p* < 0.001).

To address our first study aim, we conducted bivariate analyses examining average perceived effectiveness ratings of pictorial vs. text-only health warning labels. To address our second study aim, factor analyses using Promax rotation were conducted for Set A and for Set B pictorial HWLs, respectively. We used eigenvalues of greater than 1 as the criteria for number of factors. Then, we examined the content and internal consistency of the factors. Findings for Set A indicated one factor. Findings for Set B indicated two factors. After examining the content across Set A and Set B, all HWLs in Set A were labeled “benign” and half of the HWLs in Set B were also labeled “benign”; the remaining three HWLs in Set B were labeled “gruesome.” (These findings are described more fully below in the Results section.) 

To examine our third research aim, we conducted regression analyses. Given the different factor structures for Set A vs. Set B, we approached subsequent analyses of these two sets differently. For Set A, which involved only one factor (“benign”), we conducted linear regression to identify correlates of perceived effectiveness of the HWLs. For Set B, which included two factors (“benign” and “gruesome”), we first examined the proportion who perceived gruesome HWLs as more effective than benign (43.8%, n = 238), who perceived gruesome and benign HWLs as equally effective (43.4%, n = 236), and who perceived the benign as more effective than gruesome (12.9%, n = 70). Based on these findings, we conducted multinomial logistic regression analyses examining differences among participants who, on average, rated gruesome as more effective or benign as more effective relative to no difference. Our inclusion of potential predictors was based on the aforementioned literature regarding well-documented sociodemographic and tobacco-use related factors related to reactions to HWLs. Thus, for each set of analyses, we developed models among all participants in each set (to maximize power) and among smokers in each set, respectively. We forced the sociodemographic factors and current smoking status for analyses among all participants, and sociodemographic factors and tobacco use related factors for analyses among smokers.

## 3. Results

### 3.1. Perceived Effectiveness of Pictorial versus Text-Only HWLs

Table 1 shows that all pictorial HWLs were rated as more effective than text-only HWLs (*p*’s < 0.001), with the exceptions of the HWLs with the messaging: “Smoking can cause a slow and painful death” (*p* = 0.129) and “Smoking is highly addictive—don’t start” (*p* = 0.271).

### 3.2. Factor Analysis Examining Themes Emerging from Pictorial HWLs

Factor analysis of Set A HWLs identified one factor (“benign”), which accounted for 79.4% of the variance. Cronbach’s alphas for this factor was 0.95. Factor analysis of Set B HWLs identified two factors (see Table 2): “gruesome” and “benign”. These two factors accounted for 88.1% of the variance. Cronbach’s alphas for each factor were 0.91 and 0.94, respectively. Table 2 indicates that, while all gruesome pictorial HWLs were perceived as more effective than text-only HWLs, the two pictorial HWLs that were not perceived to be more effective than text-only HWLs were categorized as benign.

### 3.3. Correlates of Perceived Effectiveness of Benign HWLs (Set A)

Table 2 shows linear regression results examining correlates of perceived effectiveness of Set A benign HWLs. Among *all participants,* correlates with greater perceived effectiveness of benign HWLs included being female (*p* < 0.001), living in a rural setting (*p* = 0.001), not having children in the home (*p* = 0.038), and being a nonsmoker (*p* = 0.005; Adjusted R-Square = 0.104). Among *current smokers*, correlates with greater perceived effectiveness of benign HWLs included being female (*p* = 0.014), not being married/living with a partner (*p* = 0.045), having more close friends who smoke (*p* = 0.013), and rating quitting smoking as more important (*p* = 0.009; Adjusted R-Square = 0.107).

### 3.4. Correlates of Perceived Effectiveness of Gruesome Versus Benign HWLs (Set B)

Table 3 shows results of the multinomial logistic regression examining correlates of perceiving either gruesome or benign HWLs as more effective on average compared to those reporting no difference (referent group). Among *all participants,* participants rating benign HWLs as more effective had fewer friends who smoked (*p* = 0.019) and had a higher monthly household income (*p* = 0.031). No significant predictors of rating gruesome HWLs as more effective were identified (Nagelkerke R-Squared = 0.067). Among *current smokers*, compared to those rating the HWLs as equally effective, those reporting benign HWLs as more effective had fewer friends who smoked (*p* = 0.013) and rated quitting smoking to be more important (*p* = 0.006). Finally, compared to those rating the HWLs as equally effective, those reporting gruesome HWLs as more effective had a lower household income per month (*p* = 0.003; Nagelkerke R-Square = 0.497).

## 4. Discussion

The current study of Georgian adults examined how individual characteristics relate to perceived effectiveness of various cigarette HWL messaging strategies. Key findings were that not all pictorial messaging strategies were perceived equally effective. Specifically, only some benign HWLs were rated as more effective than text-only HWLs, aligning with the prior research indicating that images provoking little emotional reaction are less effective in increasing risk perceptions of smoking compared to text-only labels [41]. However, not all participants perceived gruesome images as more effective than benign images—in fact, a small proportion of participants perceived benign images as more effective, which aligns with prior research suggesting that gruesome images should not be solely relied upon [33].

These findings highlight the relevance of the ELM and the complexities in addressing tobacco use via pictorial HWLs across segments of the population with differing sociodemographic and tobacco use characteristics. Analyses regarding Set A pictorial HWLs indicated that females reported greater perceived effectiveness of the benign HWLs, which may reflect literature suggesting that women are more likely to consider quitting after viewing labels [31,42,43] and may be responsive to a broader range of pictorial HWLs, particularly those not using gruesome imagery [33]. It is important to note that two of these HWLs may be particularly relevant to women, one regarding the impact on fetuses and on appearance [12,32]. The finding that living in a rural area was associated with greater perceived effectiveness of these messages may reflect prior lack of exposure to such messaging, thus promoting more intentional processing of the information, as prior research suggests [34].

Set A analyses also indicated that being a nonsmoker was associated with greater perceived effectiveness of these images, which may reflect ELM’s notion that central processing is more likely to occur when one is faced with information congruent with one’s own beliefs, behaviors, or both [20]. Among smokers, additional correlates included being unmarried, having fewer friends who smoke, and higher self-reported importance of quitting. The findings regard being unmarried and not having children are difficult to interpret, but may reflect openness to cessation messaging as a result of fewer social influences providing prior pressure to quit smoking, thus leaving one open to central processing [20]. Analyses of Set B pictorial HWLs also found that correlates of perceiving benign HWLs as more effective included fewer friends who smoke and higher importance of quitting. Similar to prior research, those who may have fewer barriers to cessation (e.g., fewer social influences) and those who are more motivated to quit smoking may be more open to a range of pictorial HWL messaging strategies [33].

Among Set B pictorial HWLs, an additional correlate of perceiving benign HWLs more effectiveness was higher income, and among smokers, lower income was the only predictor of perceiving gruesome images as more effective. These findings are difficult to interpret but may suggest that those of higher socioeconomic status and potentially literacy may be more open to a range of HWL strategies, whereas those of lower income and literacy may be more impacted by gruesome imagery that exerts an emotional reaction [23].

The current study has significant implications for public health practice and research. In terms of practice, this study contributes to the body of evidence supporting the effectiveness of most pictorial HWL strategies relative to text-only HWLs. However, these findings highlight that, while gruesome imagery is largely seen as the most effective strategy for pictorial HWLs, other strategies may be equally or more effective on different segments of the populations (e.g., women, smokers motivated to quit). Thus, many strategies should be implemented and evaluated. Regarding future research implications, longitudinal studies in Georgia should further examine the effectiveness of varying HWL messaging strategies in long-term smoking prevention and cessation after the implementation of the new pictorial HWLs in January 2019. Given the tobacco epidemic among Georgian males and emerging epidemic among females, early smoking intervention among adolescents is critical. Future studies should examine various HWL messaging strategies as well as other smoking prevention messaging for this vulnerable population.

### Limitations

Study limitations include the potential lack of generalizability, the use of self-report measures, and the cross-sectional nature of this data, limiting our ability to determine the directionality of the relationships documented and the number of correlates examined. Moreover, the number of HWLs and distinct strategies we were able to examine was restricted. In addition, our analyses were restricted by the number and comprehensiveness of theory-driven factors that could be examined in relation to perceived effectiveness of differing HWL messaging strategies. Also, further analyses could examine individual HWLs to see who specifically perceived each as most effective could be conducted. Finally, this study does not assess actual impact of the HWL strategies tested here. As previously indicated, evaluating individual perceptions of and reactions to these HWL messaging strategies is an initial step in informing practice. Subsequent research could use experimental designs to evaluate distinct messaging strategies, and population-level evaluations could examine the ultimate impact of pictorial HWLs as they are implemented nationally.

## 5. Conclusions

Not all people perceive that gruesome or benign HWLs are effective, and it is crucial to understand the effectiveness in different populations. While pictorial HWLs are largely perceived as more persuasive, gruesome and only some benign HWLs outperform text-only. Some non-gruesome HWLs are perceived as equally persuasive, while a minority perceived that they are more effective than gruesome HWLs. In particular, non-gruesome HWLs may be more effective for smokers motivated to quit. Future research should examine how ELM could be applied as a framework to inform future tobacco prevention messaging strategies, as well as how significant social factors influence the effectiveness of various types of HWL messaging and their effects.

## Figures and Tables

**Table 1:** Comparison of persuasiveness of graphic versus text-based messages.

Message	Text M (SD)	Pictorial M (SD)		Message	Text M (SD)	Pictorial M (SD)	
	Set B	Set A	*p*		Set A	Set B	*p*
				***Gruesome***			
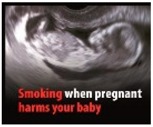	5.00 (2.76)	5.67 (2.75)	<0.001	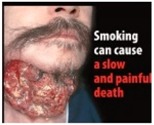	4.62 (2.62)	5.81 (2.78)	<0.001
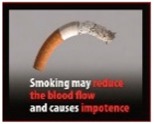	4.46 (2.65)	5.02 (2.67)	<0.001	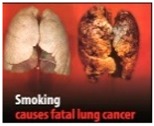	5.09 (2.63)	6.03 (2.51)	<0.001
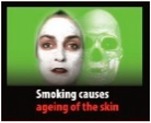	4.26 (2.65)	4.75 (2.72)	0.003	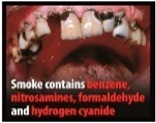	4.15 (2.63)	5.52 (2.76)	<0.001
				***Benign***			
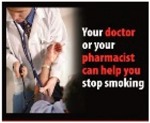	3.66 (2.68)	4.39 (2.74)	<0.001	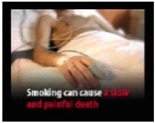	4.62 (2.62)	4.86 (2.75)	0.129
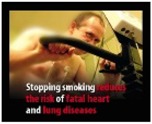	4.36 (2.61)	4.81 (2.70)	0.004	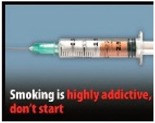	4.57 (2.68)	4.75 (2.74)	0.271
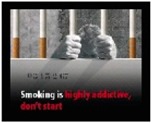	4.29 (2.66)	4.97 (2.74)	<0.001	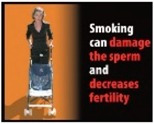	4.68 (2.66)	5.16 (2.65)	0.003

**Table 2:** Linear regression identifying correlates of reported effectiveness of benign warning labels, Set A.

Variable	Participant Characteristics	All Participants			Current Smokers		
	M (SD) or *N* (%)	Beta	95% CI	*p*	Beta	95% CI	*p*
Age, SD	42.48 (13.56)	−0.06	−0.16, 0.04	0.217	−0.063	−0.27, 0.14	0.546
Female, %	545 (49.9)	5.88	2.60, 9.17	<0.001	11.00	2.26, 19.75	0.014
Rural, %	629 (57.7)	4.69	2.00, 7.38	0.001	4.39	−0.99, 9.78	0.109
Employed full or part time, %	426 (39.2)	−2.13	−4.81, 0.54	0.118	0.97	−4.11, 6.05	0.705
# of years of education, SD	12.75 (2.85)	0.21	−0.26, 0.67	0.383	0.02	−0.80, 0.83	0.970
Income per month in Lari, SD	573.6 (630.01)	0.00	0.00, 0.00	0.089	0.00	−0.00, 0.01	0.529
Married/living with partner, %	720 (65.9)	−2.62	−5.63, 0.39	0.088	−5.82	−11.52, −0.13	0.045
Children in home, %	458 (46.5)	−2.99	−5.80, −0.17	0.038	−4.14	−9.23, 0.96	0.111
# of 5 friends who smoke, SD	2.50 (1.89)	0.10	−0.78, 0.97	0.830	2.61	0.56, 4.65	0.013
*Tobacco Use*							
Current Smoker, %	336 (30.7)	−4.87	−8.27, −1.47	0.005	-	-	-
# of days smoked, past 30, SD	20.64 (13.54)	-	-	-	0.12	−0.38, 0.62	0.633
Ave. CPD, SD	20.28 (9.54)	-	-	-	−0.07	−0.32, 0.19	0.612
Importance of quitting, SD	5.87 (3.47)	-	-	-	0.95	0.24, 1.66	0.009
Confidence in quitting, SD	4.59 (3.23)	-	-	-	0.24	−0.58, 1.06	0.568
***Adjusted R-Squared***		0.104			0.107		

# = Number; Ave. = Average.

**Table 3:** Multinomial logistic regressions comparing those who rated gruesome or benign as more effective relative to no difference (referent group), Set B.

	All Participants						Current Smokers					
	Gruesome More Effective			Benign More Effective			Gruesome More Effective			Benign More Effective		
Variable	OR	CI	*p*	OR	CI	*p*	OR	CI	*p*	OR	CI	*p*
Age	0.99	0.98, 1.01	0.360	0.99	0.97, 1.02	0.455	0.95	0.91, 1.00	0.055	0.97	0.90, 1.04	0.394
Female	0.86	0.50, 1.47	0.577	1.87	0.88, 4.00	0.104	0.25	0.04, 1.41	0.115	2.95	0.15, 56.86	0.475
Rural	1.01	0.64, 1.58	0.984	1.08	0.57, 2.10	0.567	1.75	0.55, 5.53	0.343	2.54	0.38, 17.07	0.336
Employed full or part time	1.19	0.75, 1.89	0.452	1.45	0.76, 2.78	0.258	2.73	0.83, 8.99	0.098	0.82	0.14, 5.00	0.831
# of years of education	0.95	0.88, 1.03	0.240	0.91	0.81, 1.02	0.119	1.03	0.85, 1.26	0.752	0.78	0.59, 1.03	0.074
Income per month in Lari	1.00	0.99, 1.00	0.225	1.00	1.00, 1.00	0.031	1.00	0.99, 1.00	0.003	1.00	1.00-1.00	0.228
Married/living with partner	1.16	0.70, 1.92	0.562	1.26	0.61, 2.61	0.531	0.91	0.26, 3.22	0.882	3.41	0.38, 30.32	0.271
Children in home	1.01	0.64, 1.60	0.954	1.47	0.77, 2.82	0.247	0.39	0.14, 1.11	0.076	2.44	0.50, 11.77	0.268
# of 5 friends who smoke	0.97	0.84, 1.12	0.651	0.78	0.63, 0.96	0.019	0.69	0.43, 1.09	0.107	0.45	0.24, 0.84	0.013
*Tobacco Use*												
Current smoker	1.00	0.57, 1.80	0.969	0.80	0.35, 1.81	0.587	-	-	-	-	-	-
# days smoked, past 30	-	-	-	-	-	-	1.01	0.93, 1.10	0.758	1.10	0.92, 1.23	0.428
Ave. CPD												
Importance of quitting	-	-	-	-	-	-	1.02	0.89, 1.18	0.745	1.68	1.16, 2.42	0.006
Confidence in quitting	-	-	-	-	-	-	0.98	0.84, 1.16	0.840	1.08	0.81, 1.43	0.602
***Nagelkerke R-Squared***												

# = Number; Ave. = Average.
